# Therapeutic efficacy of chloroquine plus primaquine in the treatment of uncomplicated *P.vivax* in Shecha Health Center, South Ethiopia

**DOI:** 10.1016/j.parepi.2026.e00554

**Published:** 2026-10-08

**Authors:** Dinka Dugassa, Busha Gamachu, Hussein Mohammed, Yonas Wuletaw, Geremew Tasew, Getachew Tollera, Mesay Hailu, Moges Kassa, Bokretsion G. Brhane

**Affiliations:** a Department of Clinical Pharmacy, School of Pharmacy, Institute of Health Sciences, Wollega University, Nekemte, Ethiopia; b Bacterial, Parasitic and Zoonotic Diseases, Ethiopian Public Health Institute, Addis Ababa, Ethiopia

**Keywords:** Chloroquine, Efficacy, Ethiopia, *P. vivax*, Primaquine, Treatment

## Abstract

**Background:**

Malaria is one of vector-borne diseases caused by plasmodium species. *Plasmodium falciparum* and *Plasmodium vivax* have a higher prevalence in Ethiopia. To combat this illness, effective therapy is necessary. Chloroquine plus radical cure with 14 days primaquine is a first-line antimalarial treatment for *P.vivax*. However, malaria treatment in Ethiopia is significantly impeded by resistance. The purpose of this study was to determine the therapeutic efficacy of chloroquine with radical cure with 14 days of primaquine in the treatment of uncomplicated *P. vivax* in Shecha Health Center, South Ethiopia.

**Methods:**

A single-arm, prospective therapeutic efficacy trial was conducted from December 2022 to March 2023 at Shecha Health Center in south Ethiopia. The study subjects were chosen using the purposive sampling technique. The study patients were given a 25 mg/kg standard dosage of chloroquine for three days and a 0.25 mg/kg standard dose of Primaquine for fourteen days. The World Health Organization double-entry Excel sheet was utilized for Kaplan-Meier survival analysis, and the laboratory and clinical data were analyzed using the Statistical Package for Social Sciences version-26 software. *P*-values less than 0.05 were considered statistically significant. The study endpoint was adequate clinical and parasitological response on day 42.

**Results:**

Out of the 100 study participants, 90 (90.0%) completed the 42-day follow-up period. Male research participants make 57 (57%) of the total. Adults made up the majority of the study subjects 60 (60.0%). The adequate clinical and parasitological response rate was 96.7% (95% CI: 90.1–98.9%). On day three, parasitemia was cleared at 80 (82.5%) and fever at 95 (97.9%). There were no serious adverse reactions reported.

**Conclusion:**

Chloroquine plus primaquine is still an effective drug therapy for uncomplicated *P.vivax,* with rapid clearance of parasitemia, fever, improvement of hemoglobin, and clinical resolution. Therefore, chloroquine plus primaquine should continue as a first-line drug to treat uncomplicated *P.vivax* malaria in the study area.

## 1 Background

Malaria continues to be a major public health concern worldwide, with *Plasmodium vivax* increasingly recognized as an important contributor to malaria morbidity, particularly in endemic countries striving toward elimination. Unlike *P. falciparum*, *P. vivax* possesses dormant liver-stage hypnozoites capable of causing recurrent relapses weeks or months after the initial infection, thereby sustaining transmission and complicating malaria control efforts (Jameson et al., 2022; Gebrie et al., 2024; Getu et al., 2025).

According to the World Malaria Report, an estimated 263 million malaria cases were reported globally in 2023, representing an increase compared with 249 million cases recorded in 2022. In the same year, malaria was responsible for approximately 597,000 deaths worldwide, highlighting the continued global burden of the disease (World Health Organization, 2024a). Ethiopia reported 1,978,505 malaria cases 77 deaths in April, 2025 (Health Cluster Yemen, 2019).

The COVID-19 pandemic exacerbated malaria's burden, leading to 13 million additional cases and 63,000 more deaths between 2020 and 2021 (World Health Organization, 2021a). The WHO African Region continues to shoulder the greatest burden of malaria, accounting for approximately 95% of global malaria cases and 96% of malaria-related deaths worldwide; with children under five making up 80% of fatalities (World Health Organization, 2021b). Low- and middle-income countries disproportionately suffer, with 92% of cases and 93% of deaths occurring in these regions (Habtamu et al., 2022).

Several challenges hinder malaria elimination, including inadequate funding, conflicts in endemic areas, insecticide-resistant mosquitoes, and antimalarial drug resistance (Centers for Disease Control and Prevention, 2019). In Ethiopia, malaria transmission is characterized by the co-existence of *P. falciparum* and *P.vivax*, with *P. falciparum* accounting for approximately 60–70% of malaria cases and *P. vivax* contributing 30–40% *(*Golassa and White, 2017*).* Unlike *P. falciparum*, *P.vivax* possesses a dormant hepatic stage in the form of hypnozoites, which can reactivate and cause relapse; therefore, treatment with primaquine is essential for achieving complete parasite eradication (World Health Organization, 2023).

Globally, an estimated 72 to 390 million clinical malaria cases are attributed to *P.vivax*, representing a substantial public health burden, particularly in sub-Saharan Africa (World Health Organization, 2021b). Ethiopia reported 904, 000 confirmed *P. vivax* cases in 2023 (World Health Organization, 2023). Malaria control interventions, including the use of insecticide-treated nets, indoor residual spraying, and effective antimalarial chemotherapy, have substantially reduced malaria incidence in many endemic settings (World Health Organization, 2021a).

The standard first-line treatment for uncomplicated *P.vivax* malaria consists of chloroquine for blood-stage infection, combined with a 14-day course of primaquine to achieve radical cure (Centers for Disease Control and Prevention, 2012); however, chloroquine resistance is increasing, necessitating regional monitoring and alternative treatments (Federal Democratic Republic of Ethiopia, Ministry of Health, 2022).

In Ethiopia studies assessing chloroquine efficacy against *P. vivax* remain limited, particularly regarding its combination with primaquine (Centers for Disease Control and Prevention, 2020). Antimalarial drug resistance poses a major challenge to malaria control efforts, as it compromises treatment efficacy, increases healthcare costs, and undermines the impact of existing malaria interventions (Kalra et al., 2002). Chloroquine resistance, once the cornerstone treatment, is now widespread in Africa and other regions (Kalra et al., 2002). In Ethiopia, various reports indicate substantial chloroquine treatment failure (World Health Organization, 2019).

Early detection of drug resistance is crucial to preventing the spread of resistant malaria and sustaining elimination efforts. Strengthening surveillance, developing novel drugs, and optimizing combination therapies are key strategies in combating resistance (Rasmussen et al., 2022).

Despite the widespread use of chloroquine plus primaquine in Ethiopia, data regarding its current therapeutic efficacy remain scarce in many transmission settings, including southern Ethiopia. Therefore, this study aimed to evaluate the therapeutic efficacy of chloroquine combined with primaquine for the treatment of uncomplicated *P.vivax* malaria at Shecha Health Center, South Ethiopia.

## 2 Methods and materials

### 2.1 Study area

The study was conducted from 19 December 2022 to 13 March 2023 at Shecha Health Center, located in Arbaminch Town, South Ethiopia. Arbaminch lies within the Gamo Gofa Zone of the Southern Nations, Nationalities, and Peoples' Region (SNNPR) (Fig. 1). The study area is situated approximately 500 km south of Addis Ababa in the Great Rift Valley, at a latitude of 6°01′59.99″ N and a longitude of 37°32′60.00″ E, with an altitude of 1285 m above sea level. The area has a mean annual temperature of 29.7 °C and receives an average annual rainfall of about 700 mm. Shecha Health Centre serves an estimated catchment population of approximately 31,424 people residing in Arbaminch town and surrounding communities. Arbaminch town experiences persistent malaria transmission. Transmission occurs year-round but usually increases after the main and short rainy seasons. With two major peak transmission seasons;September to December following the main rainy season, and March to May after the short rainy season (Eligo et al., 2026). Malaria transmission in the study area is highly seasonal and unstable, with an estimated entomological inoculation rate of 71.1 infected bites per person per year (Mohammed et al., 2022). The prevalent parasite species is *P.vivax*, and the primary vector is Anopheles arabiensis. (Mohammed et al., 2022; Federal Democratic Republic of Ethiopia, Ministry of Health, 2021). Based on the country's existing malaria stratification and mapping, this study region is classified as having moderate *P. vivax* transmission (altitude range of 1000 to 1750 m). (See Fig. 2.) (See Table 1, Table 2, Table 3).

**Fig. 1:**
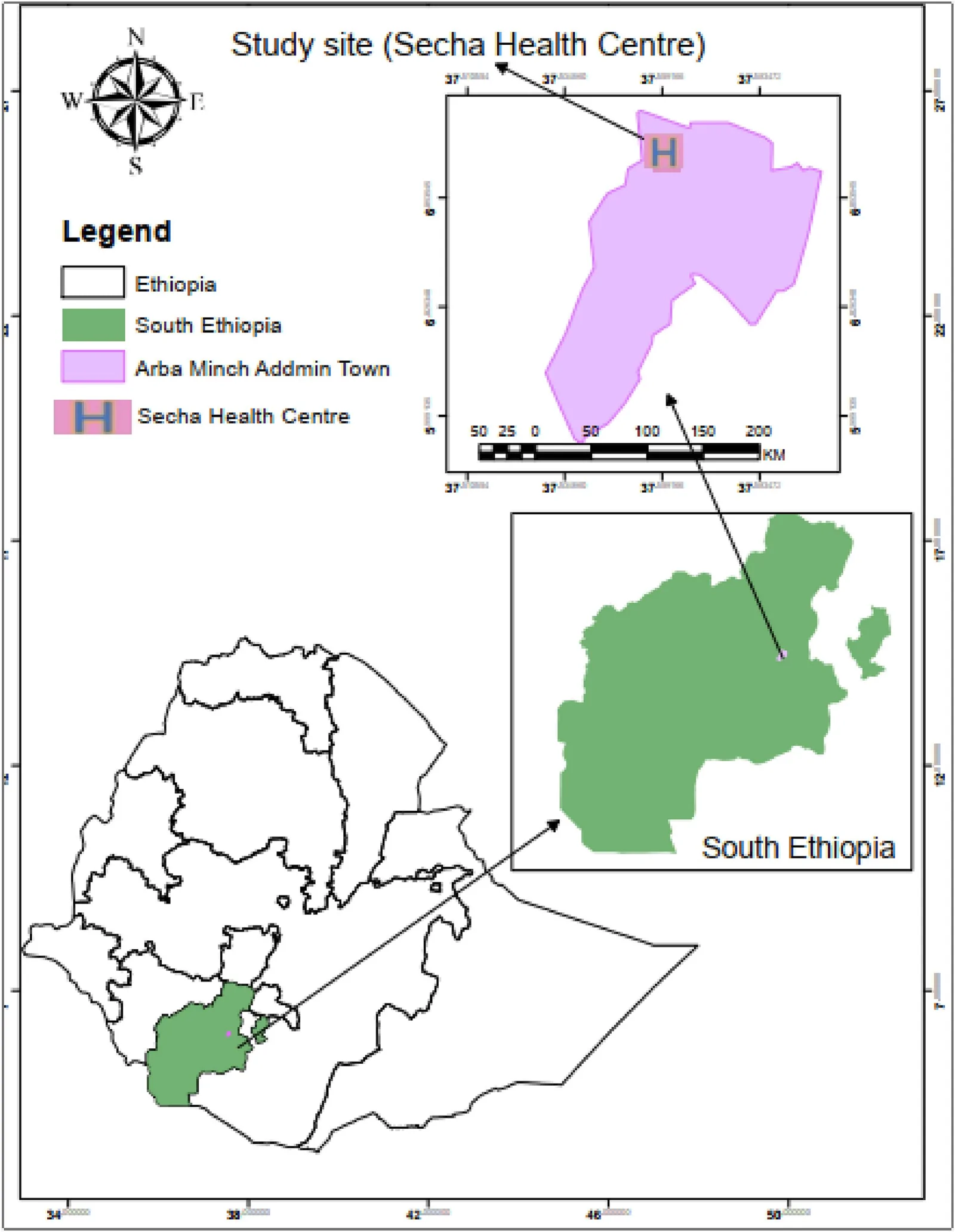
Map of the study area.

**Fig. 2:**
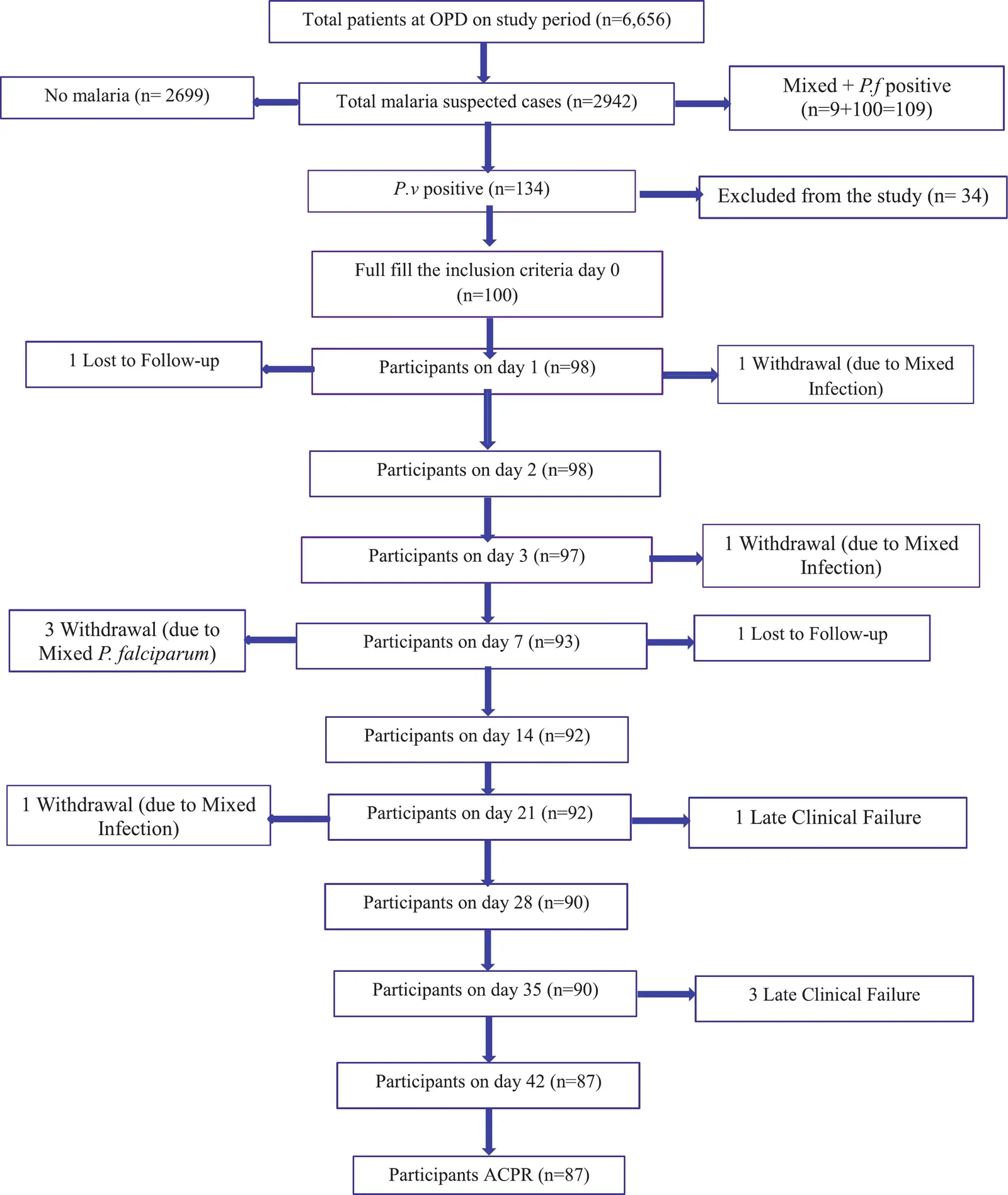
Flow chart of study participants recruitment and follow-up for chloroquine plus primaquine efficacy in Shecha Health Center, South Ethiopia.

**Table 1:** Study participants' baseline characteristics at Shecha Health Center, South Ethiopia, 2023 *n* = 100 (100%).

Variables	Frequency	Percentage
Sex		
M	57	57.0%
F	43	43.0%

Age group (years)		
Adults	60	60.0%
5 to 15	25	25.0%
under 5	15	15.0%

Age (years)		
Mean (sd)	18.57 ± (13.20)	
Range (min-max)	1.00–60.00	

Weight (kg), day 0		
Mean weight (sd)	43.9(19.9)	
Range (min-max)	9.3–87.0	

History of malaria attack		
Yes	75	75.0%
No	25	25.0%

History of antimalarial intake		
Yes	75	75.0%
No	25	25.0%

Type of antimalarial drug		
AL	55	73.3%
CQ	20	26.7%

Parasitemia (μl), day 0		
Mean (geometric) parasitemia	5140	
Range (min-max)	920–25,560	

Anemia status by gender, day 0		
Male	31	55.4%
Female	25	44.6%
Gametocyte carriage, day 0	78	78%

AL = Arthemeter- Lumefantrine, CQ = Chloroquine, sd = standard deviation.

**Table 2:** Treatment outcomes in patients with uncomplicated *P. vivax* infections treated CQ + PQ in Shecha Health Center, South Ethiopia, 2023 *n* = 100.

Category	Number	%	Lower 95%CI	Upper 95% CI
Early Treatment Failure	0	0.0	0.0	4.0
Late Clinical Failure	3	3.3	0.7	9.4
Late Parasitological Failure	0	0.0	0.0	4.0
Adequate Clinical and Parasitological Responses	87	96.7	90.6	99.3
Total patients per protocol	90			
Withdrawal	8			
Lost to Follow-up	2			
Total patients Lost to Follow-up / Withdrawal	10	10.0		
Total patients at baseline	100

**Table 3:** Hemoglobin-defined anemia severity at baseline in patients with uncomplicated *P. vivax* infections treated CQ + PQ in Shecha Health Center, South Ethiopia, 2023 *n* = 100.

Anemia category	Overall, n (%)	Male, n (%)	Female, n (%)
No anemia	44 (44.0)	19 (33.3)	25 (58.1)
Mild anemia	41 (41.0)	29 (50.9)	12 (27.9)
Moderate anemia	15 (15.0)	9 (15.8)	6 (14.0)
Severe anemia	0 (0.0)	0 (0.0)	0 (0.0)
Any anemia	56 (54.0)	38 (66.7)	18 (41.9)
Total	100 (100.0)	57 (100.0)	43 (100.0)

### 2.2 Study design

This study employed a single-arm, prospective therapeutic efficacy design to evaluate treatment outcomes among patients with uncomplicated *P.vivax* malaria. By focusing on a single arm, the research aims to provide a detailed assessment of the therapeutic interventions administered, allowing for a comprehensive understanding of their efficacy in managing this specific type of malaria. The prospective study design facilitates real-time data collection, thereby improving data accuracy and internal validity, and generating robust evidence to guide effective treatment strategies for *P.vivax* malaria.

### 2.3 Study population

Febrile patients presenting to the outpatient department of Shecha Health Center who fulfilled the inclusion criteria.

### 2.4 Eligibility criteria

#### 2.4.1 Inclusion criteria

Febrile patients presenting to the outpatient department of Shecha Health Center who fulfilled the inclusion criteria specified in the WHO protocol for antimalarial therapeutic efficacy surveillance were eligible for enrolment in the study (World Health Organization, 2009) Eligible participants were: individuals aged six months and older, who presented with fever (axillary temperature ≥ 37.5 °C) or history of fever during the 48 h before recruitment, had microscopically confirmed *P.vivax* mono-infection, and an asexual parasite density of at least 250 parasites/μL, participants were required to reside within the catchment area of the health facility, defined as within a 10-km radius of the health center.

#### 2.4.2 Exclusion criteria

Participants were excluded if they were pregnant or breastfeeding; had mixed *Plasmodium* species infection (*P. falciparum* and *P. vivax*); a baseline hemoglobin level below 5.0 g/dL; inability to tolerate oral medication or persistent vomiting; known hypersensitivity to chloroquine or primaquine; clinical signs of severe malaria; presence of chronic illness or severe malnutrition; or evidence of non-malarial febrile conditions, including otitis media, tonsillitis, measles, acute lower respiratory tract infection, or severe diarrhea accompanied by dehydration.

#### 2.4.3 Sample size determination

The study was conducted in accordance with the World Health Organization (WHO) 2009 guidelines for the surveillance of antimalarial drug therapeutic efficacy (World Health Organization, 2009); the sample size was calculated using the single population proportion formula, assuming a 95% confidence level and a 5% margin of error. A minimum of 73 participants was required, which was increased by 20% to accommodate potential loss to follow-up and withdrawals over the 42-day study period, resulting in a target enrollment of 100 participants.

#### 2.4.4 Laboratory procedures

At each follow-up visit, both thick and thin blood smears were prepared on a single slide for parasite detection and species identification, respectively. Two blood smears were obtained from each participant at every visit. The initial screening smear was stained with 10% Giemsa for 10–15 min, while a second smear was stained with 3% Giemsa for 30 min and independently examined by two experienced laboratory technicians at the health center. In cases of discordant results between the two readings, a third independent examination was performed to reach a final diagnosis.

Parasite density was recorded for all positive blood smears. Asexual parasitemia was quantified by counting the number of asexual parasites per 200 white blood cells (WBCs) and expressed as parasites per microliter of blood, assuming a standard WBC count of 8000/μL. Gametocyte density was determined by counting against 500 WBCs. A smear was considered negative only after examining at least 200 oil-immersion high-power fields on the thick film. To ensure microscopy quality and accuracy, all blood slides were cross-validated by a WHO-accredited microscopist at the Ethiopian Public Health Institute.

#### 2.4.5 Hematological procedures

Hemoglobin concentrations were measured using a portable spectrophotometer (HemoCue®, Ängelholm, Sweden) on Days 0, 14, 28, and 42. Female participants aged 12 years and older were screened for pregnancy at the time of enrollment.

#### 2.4.6 Anemia classification

Anemia was defined and classified according to the World Health Organization (WHO) 2024 guideline on hemoglobin cutoffs for defining anemia. For children aged 6–23 months, anemia was defined as a hemoglobin (Hb) concentration < 10.5 g/dL, with mild, moderate, and severe anemia classified as 9.5–10.4 g/dL, 7.0–9.4 g/dL, and < 7.0 g/dL, respectively. For children aged 24–59 months, anemia was defined as Hb <11.0 g/dL, with mild, moderate, and severe anemia classified as 10.0–10.9 g/dL, 7.0–9.9 g/dL, and < 7.0 g/dL, respectively. For adults aged 15–65 years, anemia was defined as Hb <13.0 g/dL in males and < 12.0 g/dL in non-pregnant females; mild anemia was classified as 11.0–12.9 g/dL in males and 11.0–11.9 g/dL in females, moderate anemia as 8.0–10.9 g/dL in both sexes, and severe anemia as <8.0 g/dL in both sexes. Participants with Hb concentrations at or above the corresponding age- and sex-specific thresholds were considered non-anemic. These predefined WHO thresholds were applied consistently throughout the study to ensure standardized, reproducible, and internationally comparable classification of anemia.

### 2.5 Treatment and follow-up

Eligible participants were treated with a standard three-day course of chloroquine (CQ) followed by a 14-day course of primaquine (PQ). Chloroquine administration was fully supervised through directly observed therapy, whereas primaquine was administered under semi-supervised conditions. All drug dosages were prescribed in strict accordance with the Ethiopian national malaria treatment guidelines (Federal Democratic Republic of Ethiopia, Ministry of Health, 2022).

Chloroquine was administered at a total dose of 25 mg base/kg over three consecutive days (10 mg base/kg on Days 0 and 1, and 5 mg base/kg on Day 2), using 250 mg chloroquine tablets. Primaquine was given concurrently at a dose of 0.25 mg base/kg once daily for 14 days, using 7.5 mg primaquine tablets. Drug administration within the health facility was supervised by trained clinical staff. Following each dose, participants were observed for 60 min to monitor for vomiting or other adverse reactions. Patients who vomited within 30 min of drug intake were re-dosed with the full amount, whereas those who vomited between 30 and 60 min received a half dose. Participants who vomited on two occasions were withdrawn from the study and referred to a higher-level health facility for appropriate clinical management (World Health Organization, 2009). All study medications were supplied by the Ethiopian Pharmaceutical Supply Service. Chloroquine 250 mg tablets were manufactured by IPCA Laboratories Ltd., India (Batch No. L8080013; Expiry Date: April 2023). Primaquine 7.5 mg tablets were manufactured by Remedica Ltd., Limassol, Cyprus (Batch No. 92147).

Participants were followed daily for the first three days following treatment initiation (Day 0) and subsequently at weekly intervals on Days 7, 14, 21, 28, 35, and 42. Comprehensive clinical evaluations and laboratory assessments were conducted at each scheduled follow-up visit. Additional unscheduled visits were arranged when participants developed new or recurrent symptoms. Adverse events were defined according to World Health Organization (WHO) guidelines for antimalarial therapeutic efficacy monitoring and were systematically documented at every follow-up visit (World Health Organization, 2009).

Fever and parasite clearance were assessed during the first seven days of follow-up. Early treatment failure (ETF) and early parasitological failure (EPF) were defined in accordance with the World Health Organization (WHO) 2009 guidelines as outcomes occurring within the first three days following treatment initiation. Late clinical failure (LCF) and late parasitological failure (LPF) were defined as the reappearance of parasitemia between Days 4 and 28 (or up to Day 42), with or without concomitant fever, respectively, provided that none of the criteria for ETF or EPF had been fulfilled earlier.

### 2.6 Statistical analysis

Study endpoints were categorized as primary and secondary outcomes. The primary endpoint was the overall therapeutic efficacy of chloroquine plus primaquine (CQ + PQ) at Day 42, measured as the proportion of participants achieving an adequate clinical and parasitological response (ACPR). Secondary endpoints included the incidence of adverse events, rates of fever and parasite clearance, and changes in hemoglobin levels over the follow-up period.

Data were double-entered into the World Health Organization (WHO) standardized Excel database designed for therapeutic efficacy studies and subsequently analyzed using IBM SPSS Statistics software (version 26). Descriptive statistics, including means, standard deviations, and proportions, were generated. In accordance with WHO guidelines, the adequate clinical and parasitological response (ACPR) at Day 42 was estimated using the Kaplan–Meier cumulative risk method (World Health Organization, 2009).

### 2.7 Ethical consideration

The study protocol was reviewed and approved by the Institutional Review Board (IRB) of the Ethiopian Public Health Institute (EPHI). Written informed consent was obtained from all participants aged 18 years and older. For participants younger than 18 years, written informed consent was obtained from parents or legal guardians, and written informed assent was additionally secured from children aged 12–17 years.

## 3 Results

### 3.1 Baseline characteristics

During the study period, 6656 patients attended the outpatient department of Shecha Health Center. Among these, 2942 (44.2%) were suspected malaria cases, and 134 (4.5%) were microscopically confirmed as *P.vivax* infections. Of the confirmed cases, 100 patients (74.6%) with *P. vivax* mono-infection who fulfilled the inclusion criteria were enrolled in the study. Male participants constituted the majority, with a male-to-female ratio of approximately 1.3:1 (57.0% vs. 43.0%). The age of participants ranged from 1 to 60 years, with a mean age of 18.57 ± 13.2 years, and 60 (60.0%) of the participants were adults. Body weight ranged from 9.3 to 87 kg.

Among the enrolled participants, 40 (40.0%) reported owning insecticide-treated bed nets, of whom 27 (67.5%) had slept under a net on the night preceding enrollment. Seventy-five (75.0%) of participants reported a previous history of malaria, and all had received antimalarial treatment. Of the 100 participants enrolled, 90 (90.0%) successfully completed the 42-day follow-up period. No serious adverse drug reactions were observed following treatment with chloroquine plus primaquine (CQ + PQ).

At enrollment, 97 (97.0%) participants presented with fever, while the remaining participants reported a history of fever within the preceding 48 h. The mean baseline axillary temperature was 38.64 ± 0.9 °C (range: 36.5–40.5 °C), with the highest mean temperature recorded among children under five years of age (39.3 ± 0.1 °C). The most commonly reported clinical symptoms on Day 0 were headache, vomiting, and nausea.

At baseline, the mean hemoglobin concentration was 12.66 ± 1.75 g/dL (range: 8.9–17.8 g/dL), with 56 (56.0%) of participants classified as anemic.

The mean asexual parasite density at enrollment was 5140 parasites/μL, ranging from 920 to 25,560 parasites/μL. In addition, gametocytes were detected in 78 (78.0%) of participants at baseline.

#### 3.1.1 Therapeutic efficacy of chloroquine plus primaquine

Based on the per-protocol analysis of the 90 (90.0%) participants who completed the full follow-up period, 87 (96.7%) achieved an adequate clinical and parasitological response (ACPR).

Kaplan–Meier survival analysis over the 42-day follow-up period showed a cure rate of 96.7% (95% CI: 90.6–99.3%) for chloroquine plus primaquine (Fig. 3). (See Fig. 4.)

**Fig. 3:**
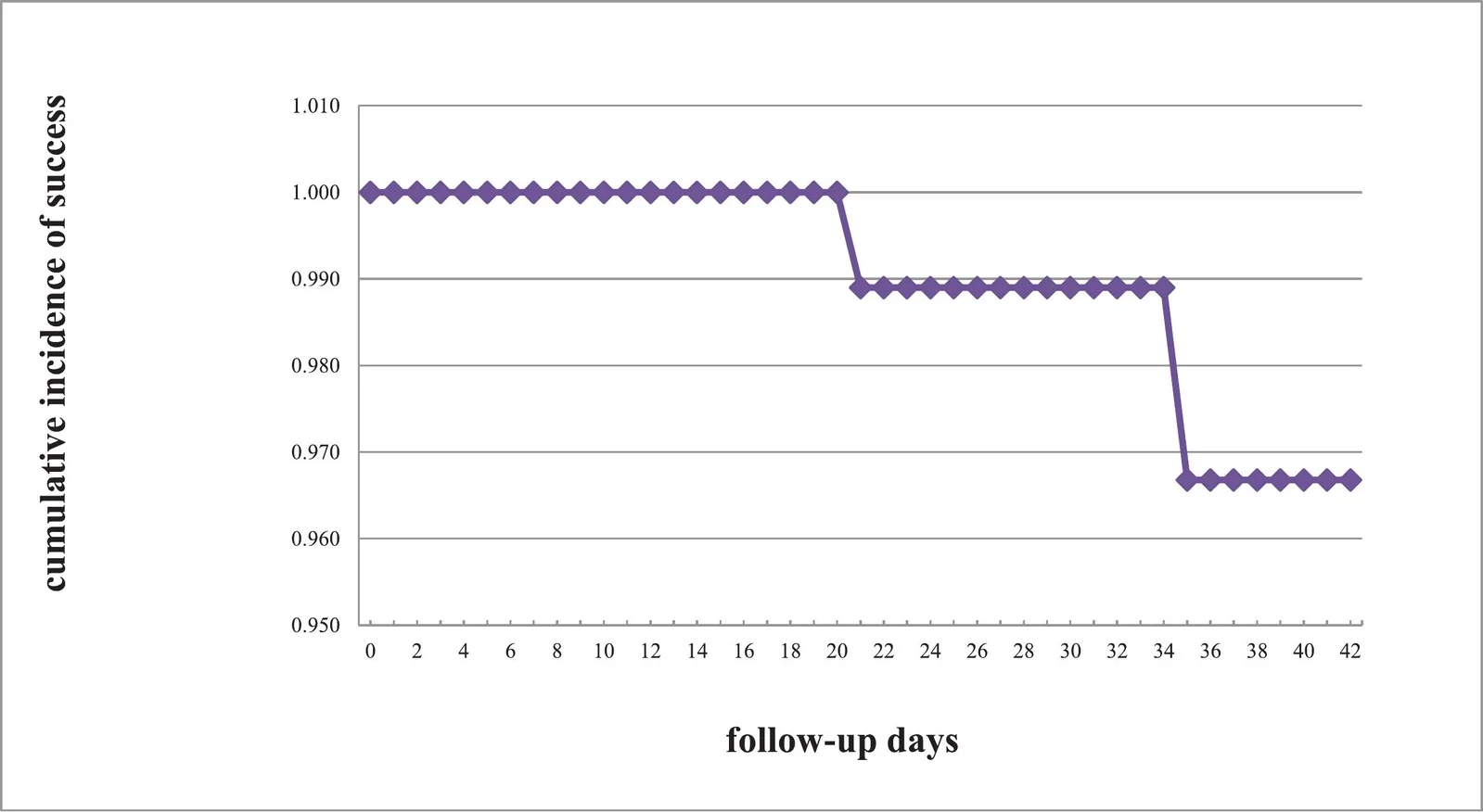
Kaplan-Meier survival curve showing treatment results throughout the course of the 42-day follow-up period at Shecha Health Center in southern Ethiopia between December 2022 and March 2023.

**Fig. 4:**
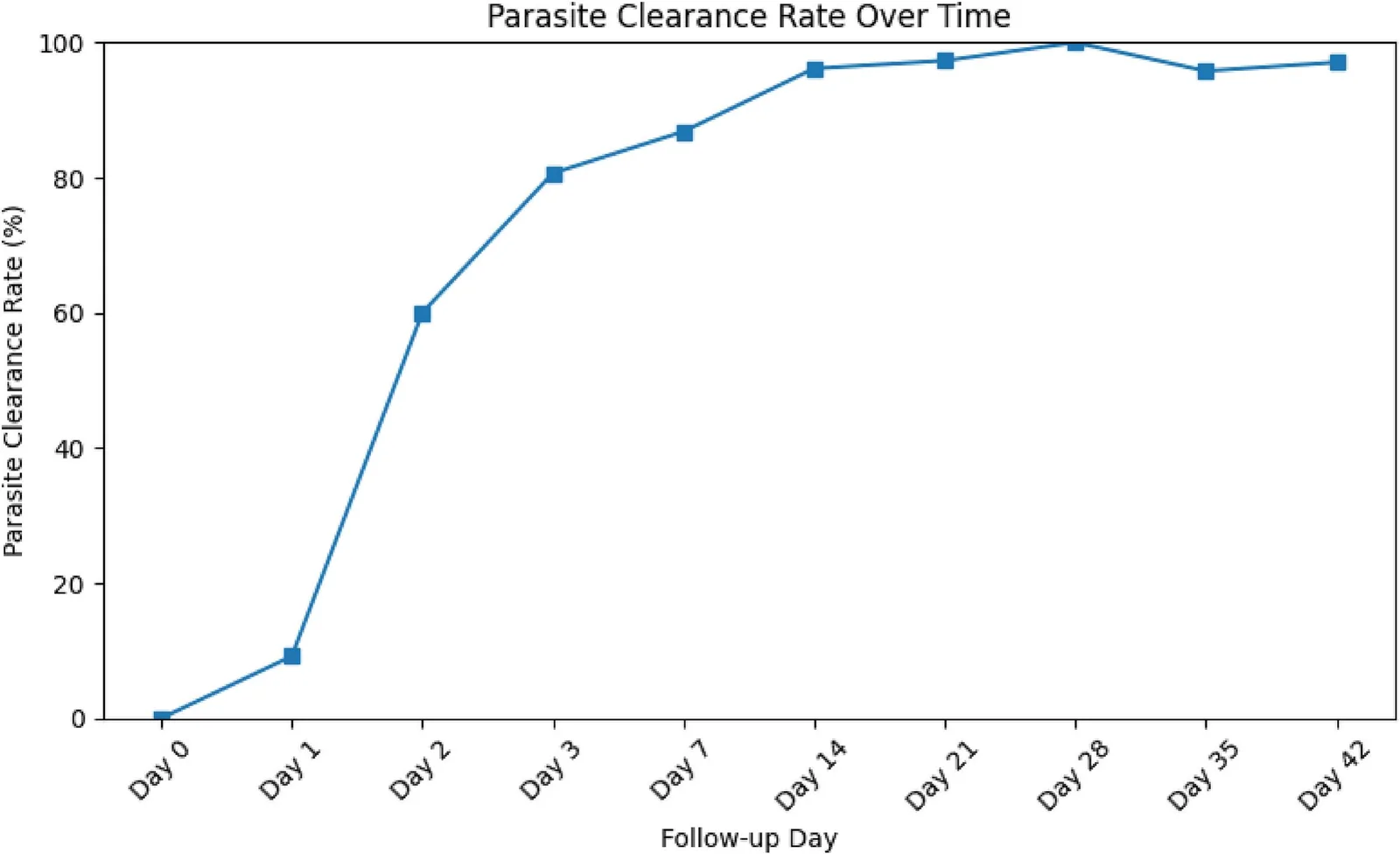
Parasite clearance rate throughout the course of the 42-day follow-up period at Shecha Health Center in southern Ethiopia between December 2022 and March 2023.

#### 3.1.2 Parasite clearance

Per-protocol analysis showed that 80 (82.5%) of participants were free of parasitemia by Day 3 of follow-up. Among the three participants who experienced treatment failure, one demonstrated an increase in parasite density relative to baseline, whereas the remaining two had reduced parasitemia levels compared with their baseline measurements.

#### 3.1.3 Fever clearance

By Day 3 of follow-up, fever had resolved in 95 (97.9%) of participants. Fever clearance was significantly associated with parasite clearance on Day 3 (*p* = 0.001). However, a small proportion of participants experienced recurrent or persistent fever during subsequent follow-up visits.

#### 3.1.4 Gametocyte clearance

At baseline (Day 0), the majority of participants had detectable gametocytes, whereas substantial clearance was observed by Day 3 and complete clearance was achieved among nearly all participants during later follow-up visits. The mean gametocyte density also progressively declined over time, indicating good therapeutic response and effective parasite clearance.

### 3.2 Hemoglobin

At baseline, 56.0% (56/100) of participants met the WHO 2024 criteria for anemia (World Health Organization, 2024b). Mild anemia was the predominant severity category, affecting 41.0% (41/100) of participants, while 15.0% (15/100) had moderate anemia. No participant was classified as having severe anemia. Anemia was more prevalent among males than females (66.7% vs. 41.9%). Among males, 50.9% had mild anemia and 15.8% had moderate anemia, compared with 27.9% and 14.0%, respectively, among females.

The prevalence of anemia showed a progressive decline during follow-up, decreasing to 45 participants by Day 14, 10 participants by Day 28, and only 6 participants by Day 42. In parallel, mean hemoglobin concentration increased progressively from 11.8 ± 1.97 g/dL at baseline (Day 0) to 11.9 ± 1.40 g/dL on Day 14, 12.5 ± 1.30 g/dL on Day 28, and 12.7 ± 1.27 g/dL on Day 42, indicating a gradual improvement in hematological status throughout the follow-up period (Fig. 5).

**Fig. 5:**
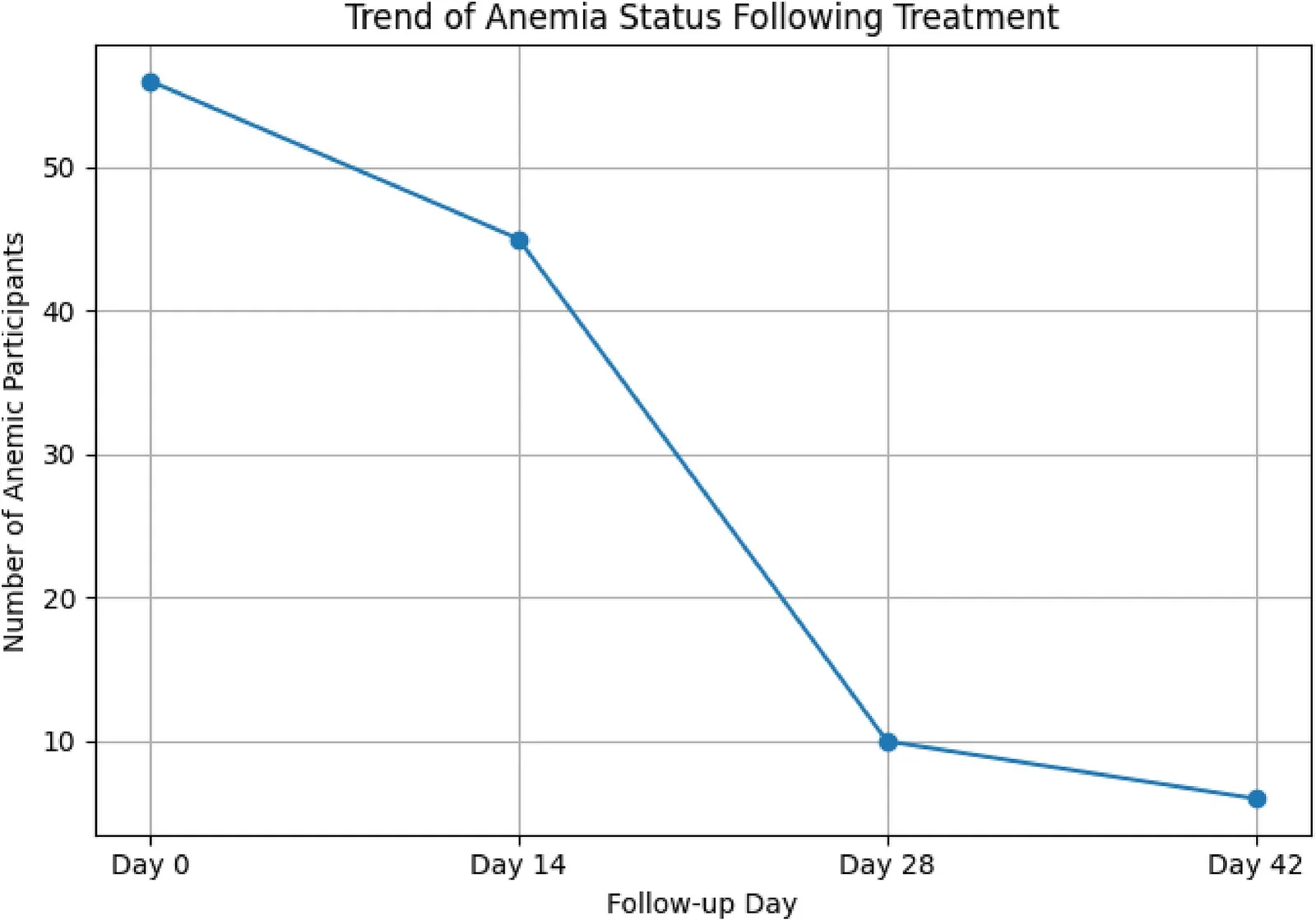
Trend of anemia status throughout the course of the 42-day follow-up period at Shecha Health Center in southern Ethiopia between December 2022 and March 2023.

### 3.3 Adverse events

At baseline, the most frequently reported clinical signs and symptoms among participants were fever (97.0%), headache (88.0%), shivering (23.0%), and vomiting (13.0%). Following treatment, reported adverse events were generally mild and included nausea (9.0%), dizziness (5.0%), confusion (3.0%), cough (3.0%), and abdominal pain (2.0%).

## 4 Discussion

The development and spread of antimalarial medication resistance pose a growing danger to the management, prevention, and control of malaria. In Ethiopia, the first-line treatment for uncomplicated *P.vivax* malaria has historically been chloroquine plus primaquine (CQ + PQ). However, questions about the long-term efficacy of this treatment plan have been highlighted by mounting evidence of decreased susceptibility and developing resistance to CQ + PQ in a number of countries (Awab et al., 2017). Regular monitoring of antimalarial drug therapeutic efficacy is essential for early detection and timely response to the emergence of resistance to existing antimalarial treatments. Accordingly, this study aimed to evaluate the therapeutic efficacy of chloroquine plus primaquine (CQ + PQ) in the treatment of patients with *P.vivax* malaria at Shecha Health Center.

In this single-arm, prospective therapeutic efficacy study, the unadjusted cure rate of chloroquine plus primaquine (CQ + PQ) was 98.8% (95% CI: 95.3–100%) at Day 28. By Day 42, the unadjusted cure rate was 96.7% (95% CI: 90.1–98.9%), while the per-protocol analysis showed a 100% cure rate. By Day 3 of follow-up, the rates of parasite clearance, gametocyte clearance, and fever clearance were 82.5%, 100%, and 97.9%, respectively. Furthermore, mean hemoglobin concentration increased significantly over the follow-up period, rising from 11.9 ± 1.4 g/dL at baseline to 13.4 g/dL on Day 42 (*p* < 0.002).

This study demonstrated the high therapeutic efficacy of chloroquine plus primaquine (CQ + PQ), with a cure rate of 98.8% at 28 days of follow-up, highlighting the effectiveness of this regimen for treating *P.vivax* malaria. At 42 days post-treatment, the cure rate of 96.7% further confirm the ability of CQ + PQ to achieve sustained parasite clearance. However, it is important to note that standardized molecular markers capable of reliably distinguishing reinfection from recrudescence in *P. vivax* are currently unavailable, representing a limitation not only of the present study but also of *P. vivax* therapeutic efficacy research more broadly.

In the present study, extending the follow-up period to 42 days resulted in a reduction of the observed cure rate to 96.7%. This finding suggests that residual therapeutic concentrations of chloroquine may persist in the bloodstream for approximately 21–35 days after treatment initiation, suppressing blood-stage parasitemia to levels below the threshold of microscopic detection (Lee et al., 2008). These findings are consistent with results reported in studies conducted in Brazil (97.8%) (Ladeia-Andrade et al., 2019), Ranong, Colombia (98.1%) (Mesa-Echeverry et al., 2019), and Northeast, Myanmar (98.6%) (Soe et al., 2022). The observed similarities in cure rates may be attributed to comparable study designs, participant characteristics, treatment regimens, sample sizes, and outcome assessment methods.

After Day 28, circulating chloroquine concentrations may decline, as evidenced by the fact that 66.7% of treatment failures in this study occurred beyond this time point. This reduction in drug levels may allow previously suppressed blood-stage parasites to proliferate. Nevertheless, the frequency of adequate clinical and parasitological response (ACPR) observed in the present study prior to Per-protocol analysis (96.7%) was substantially higher than that reported in a study conducted at Kola Shele, which documented an ACPR of 89.1% (Mekonnen et al., 2023). This difference may be attributable to variations in sample size and the socio-demographic characteristics of the study populations.

In contrast to the present findings, higher treatment failure rates for chloroquine plus primaquine (CQ + PQ) have been reported at various follow-up intervals in different regions of Ethiopia, including a rate of 18.2% at the current study site two years ago (Assefa et al., 2022), Debrezeit and Nazareth towns, 7.5% (Yohannes et al., 2011), and elsewhere Madagascar 5.1% (Barnadas et al., 2008). This may be because those study finding is only on chloroquine alone while in this study primaquine is used to eradicate the dormant stage of *P.vivax* and difference in sample size, study setting and follow-up period. Relapse, malabsorption of drug, reinfection, other patient factors (i.e. unnecessary use of antimalarial drugs, poor compliance to dosing regimen) and drug-related factors(i.e. poor quality drugs and monotherapies) could be contributing factors to treatment failure of *P.vivax (*Bloland*, 2001**)*.

Kaplan–Meier analysis in the present study indicated a treatment failure rate of 3.3% (95% CI: 1.1%–9.9%) prior to Per-protocol analysis, with no treatment failures observed in the per-protocol analysis. These results are comparable to findings from Yala and Sakaeo in Colombia (0% each), southern India (0.8%), and Kolkata (0%). In contrast, the observed failure rate in this study was lower than those reported in other regions of Ethiopia, including Debre Zeit (7.5%, 95% CI: 2.9%–18.9%) and Arbaminch (10.9%, 95% CI: 5.8%–19.9%) (Mekonnen et al., 2023; Yohannes et al., 2011; Vijaykadga et al., 2004; Seifu et al., 2017; Getachew et al., 2015). In this study, some treatment failures were observed after 28 days of follow-up. These differences may be attributed to variations in baseline characteristics of participants, sample size, study settings, and the duration of follow-up. A longer follow-up period (42 days) increases the likelihood of detecting reinfection compared with shorter follow-up (28 days). Additionally, late treatment failures may reflect relapse or emerging resistance of P.vivax to chloroquine plus primaquine (CQ + PQ) therapy.

In the present study, the parasite clearance rate by Day 3 was 82.5%, which is comparable to findings reported in India, where a clearance rate of 95.5% was observed (Ganguly et al., 2013), and Singapore 93.2% (Subramony et al., 2016). This similarity may be attributable to comparable participant characteristics, sample sizes, and study designs. However, the Day 3 parasite clearance rate in the present study (82.5%) was slightly lower than that reported in northeastern Myanmar, where a clearance rate of 98.6% was observed (Xu et al., 2020). This difference may be attributed to variations in participant characteristics, sample size, and the presence of chloroquine-resistant strains of *P.vivax* in the geographical region. Several factors can influence parasite clearance (White, 2011), including host immunity (McGregor, 1964), drug efficacy (White, 2017), and drug absorption (Ducharme and Farinotti, 1996).

In the present study, 60.0% of participants were over 15 years of age (adults), suggesting that adults may have higher immunity compared with children and may be less affected by potential reductions in CQ + PQ efficacy. Additionally, drug absorption, which can influence parasite clearance, may have been enhanced in this study because participants consumed biscuits prior to drug administration. Food intake before medication is known to improve the absorption of antimalarial drugs, potentially contributing to better therapeutic outcomes (World Health Organization, 2009; Ducharme and Farinotti, 1996). In this study, 17.5% of participants (95% CI: 2.5–9.6) remained parasitemic on Day 3, which is higher than the 15% positivity reported in a study conducted in Myanmar (Soe et al., 2022). This difference may be attributed to variations in participant characteristics, sample size, and geographic location.

In the present study, 75% of participants reported a prior malaria infection. Repeated malaria exposure is known to contribute to the development of partial immunity in the host (White, 2017); this may contribute to enhanced parasite clearance. The observed difference could also be related to the use of combination therapy (CQ + PQ) in the present study, which may exert a synergistic effect on parasite elimination; however, this hypothesis warrants further comparative investigation.

The complete gametocyte clearance observed by Day 3 (100%) is a notable finding, highlighting the potential of the CQ + PQ regimen to interrupt malaria transmission. Additionally, the high fever clearance rate of 97.9% by Day 3 underscores the effectiveness of this treatment in alleviating the clinical symptoms associated with *P.vivax* infection.

In this study, the gametocyte clearance rate reached 100% by Day 3, which is higher than that reported in other studies like 96.5% in China (Xu et al., 2020), Brazil 88.5% (Soe et al., 2022). These findings are consistent with a study conducted in Shewa Robit, where 100% of participants also achieved gametocyte clearance by Day 2 (Ladeia-Andrade et al., 2019). This difference may be attributed to variations in drug efficacy or baseline gametocyte density. In the present study, the mean gametocyte count at enrollment was 1309.1, whereas baseline gametocyte densities were not clearly reported in other studies.

In the present study, the fever clearance rate among participants was 97.9% by Day 3, consistent with findings reported in Thailand 98% (Subramony et al., 2016). However, participants in the present study exhibited a higher fever clearance rate compared with findings from Singapore (Belay et al., 2023) and Shewa Robit (Ladeia-Andrade et al., 2019), where a rate of 91% and 90.7% were reported respectively. This difference may be attributed to geographical variations.

Although previous studies have suggested an association between parasite clearance and fever resolution, the present study demonstrated a statistically significant correlation between fever clearance and parasite clearance on Day 3 (*p* = 0.001). Fever in malaria arises from the rupture of blood-stage schizonts, releasing merozoites and various toxins including glycosylphosphatidylinositol, DNA-associated hemozoin, and cholesterol–triglyceride complexes which not only facilitate the infection of new red blood cells but also trigger host anti-toxin immune responses (Anstey et al., 2009). This suggests that early parasite clearance reduces the likelihood of persistent fever. In the present study, the rapid clearance of parasites may have contributed to the high fever clearance rate. However, the fever clearance rate observed here was slightly lower than that reported in a study conducted three years ago in Arbaminch, where 100% of participants were fever-free by Day 3, corresponding to a 100% parasite clearance rate by Day 2 (Mekonnen et al., 2023).

The observed increase in mean hemoglobin levels from 12.7 g/dL to 13.4 g/dL represents a notable improvement, potentially reflecting the restoration of hemoglobin following effective treatment. The high baseline prevalence of anemia (56.0%) highlights the impact of *P.vivax* infection on hemoglobin levels and underscores the importance of successful antimalarial therapy in mitigating these effects.

In the present study, paired *t*-test analysis revealed a significant increase in mean hemoglobin levels from 11.9 ± 1.4 g/dL at baseline to 13.4 g/dL at Day 42 (*p* < 0.002). This finding is consistent with results reported in a previous study conducted in Arbaminch town (Mekonnen et al., 2023) 11.8 g/dL to 13.4 g/dL (*P* < 0.001) between day 0 and day 42.

However, the baseline prevalence of anemia among participants in the present study (56.0%) was higher than that reported in studies conducted in Shewa Robit (27.9%) (Ladeia-Andrade et al., 2019), and Southern Ethiopia (18.5%) (Getachew et al., 2015). Malaria associated anemia primarily results from the destruction of red blood cells by parasites and the immune-mediated clearance of infected erythrocytes (Collins et al., 2003).

Younger children produce lower levels of pro-erythropoietic cytokines and exhibit reduced expression of complement regulatory proteins on red blood cells, both of which are essential for effective inflammatory and immune responses (Nussenblatt et al., 2001; Waitumbi et al., 2004), These immunological factors may contribute to higher parasite densities due to less efficient parasite clearance. In the present study, a substantial proportion of participants were young children, which may partly explain the higher levels of parasitemia observed. Increased parasite burden can, in turn, lead to enhanced destruction of red blood cells by parasites and greater immune-mediated clearance of infected erythrocytes.

Although the treatment demonstrated high therapeutic efficacy, a small proportion of participants experienced adverse events, including anorexia, nausea, dizziness, and cough. While these events were generally mild and infrequent, they underscore the importance of ongoing monitoring and appropriate management of potential side effects to ensure the safety and tolerability of the chloroquine plus primaquine (CQ + PQ) regimen.

At enrollment, the most common clinical signs and symptoms among participants were fever (97%), headache (88%), shivering (23%), and vomiting (13%). Following treatment, a small number of adverse events were reported, including confusion, dizziness, cough, abdominal pain, and nausea. These adverse events are consistent with those reported in previous studies (World Health Organization, 2015). The reported adverse events included fever, headache, anorexia, nausea, vomiting, abdominal pain, diarrhea, cough, behavioral changes, and dizziness. These findings are consistent with adverse events described in the World Health Organization (WHO) 2015 guidelines (Ladeia-Andrade et al., 2019), and no serious adverse events or hypersensitivity reactions were observed throughout the entire follow-up period.

The findings of this study have important implications for clinical practice, malaria case management, and public health interventions. The high therapeutic efficacy of the CQ + PQ regimen, together with rapid parasite and gametocyte clearance and significant improvement in hemoglobin levels, highlights its effectiveness in the treatment of uncomplicated P.vivax malaria. Although adverse events were infrequent and mild, their occurrence underscores the need for continued clinical vigilance and appropriate patient management. Furthermore, these results emphasize the importance of sustained therapeutic efficacy surveillance and systematic monitoring of treatment outcomes and drug-related adverse events to support evidence-based malaria control strategies.

The therapeutic efficacy of chloroquine (CQ) combined with primaquine (PQ) for the treatment of uncomplicated *P.vivax* malaria at Shecha Health Center in Arbaminch town provides compelling evidence of the efficacy of this regimen. The study demonstrates high cure rates, rapid clearance of both parasites and gametocytes, significant improvement in hemoglobin levels, and timely resolution of clinical symptoms. In addition, the occurrence of adverse events, although limited, offers valuable insight into the safety profile of the CQ + PQ regimen and underscores the importance of continued monitoring during treatment.

Despite the important findings of this study, several limitations should be acknowledged. First, the study was conducted at a single health center with a relatively limited sample size, which may affect the generalizability of the findings to other malaria-endemic settings. Second, the single-arm study design without a comparison group limited the ability to directly compare the efficacy and safety of chloroquine plus primaquine (CQ + PQ) with alternative antimalarial regimens. In addition, adherence to the final nine days of primaquine treatment was assessed through participant self-report, which may have introduced recall or reporting bias. Furthermore, chloroquine blood concentration levels status were not evaluated, limiting assessment of the potential influence of drug exposure and host-related factors on treatment outcomes and adverse events.

## 5 Conclusion

The present study demonstrates that the combination of chloroquine and primaquine (CQ + PQ) maintains therapeutic efficacy in the treatment of uncomplicated *P.vivax* malaria. This regimen was associated with rapid clearance of parasitemia and fever, significant improvement in hemoglobin levels, and favorable overall clinical outcomes. The addition of primaquine appears to enhance treatment effectiveness, even in settings where routine glucose-6-phosphate dehydrogenase (G6PD) testing is not available. Importantly, no severe adverse events were reported during the follow-up period. These findings support the continued use of CQ + PQ as the first-line therapy for uncomplicated *P. vivax* malaria under national and regional malaria elimination programs. However, large-scale, continuous, and robust therapeutic efficacy surveillance is essential for the early detection and effective management of potential antimalarial drug resistance, both locally and nationwide.

## Author contribution

DD, BGB developed the protocol. BG, MK revised the protocol. DD and BG supervised overall the study from the conception. DD, BG, HM, YW, MK, MH, GT, GT and BGB conducted and monitored the data collection and analysis. DD developed the draft manuscript, and BG, MK, HM, YW, GT, GT, MH and BGB critically reviewed. All authors reviewed and edited the manuscript, and approved the final version for publication.

## CRediT authorship contribution statement

**Dinka Dugassa:** Writing – review & editing, Writing – original draft, Visualization, Validation, Supervision, Software, Resources, Project administration, Methodology, Investigation, Formal analysis, Data curation, Conceptualization. **Busha Gamachu:** Writing – review & editing, Writing – original draft, Visualization, Supervision, Software, Resources, Methodology, Formal analysis, Data curation, Conceptualization. **Hussein Mohammed:** Visualization, Validation, Supervision, Software, Methodology, Data curation, Conceptualization. **Yonas Wuletaw:** Visualization, Validation, Supervision, Resources, Methodology, Conceptualization. **Geremew Tasew:** Visualization, Validation, Supervision, Resources, Conceptualization. **Getachew Tollera:** Validation, Supervision, Resources, Project administration, Funding acquisition, Conceptualization. **Mesay Hailu:** Validation, Resources, Project administration, Funding acquisition, Conceptualization. **Moges Kassa:** Visualization, Validation, Supervision, Software, Resources, Project administration, Methodology, Funding acquisition, Formal analysis, Data curation, Conceptualization. **Bokretsion G. Brhane:** Writing – review & editing, Writing – original draft, Visualization, Validation, Supervision, Software, Resources, Project administration, Methodology, Investigation, Funding acquisition, Formal analysis, Data curation, Conceptualization.

## Consent for publication

Not applicable.

## Ethics approval and consent to participate

The study received ethical approval from the scientific and ethical review board of Ethiopian Public Health Institute (protocol approval number EPHIIRB-294/2022). Written informed consent was obtained from all of the study participants ≥18 years of age or by parents or guardians of children less than 18 years of age, with children 12–17 years of age providing written informed assent.

## Funding

This work was supported by the Ethiopian Public Health Institute, Ministry of Health-Ethiopia, and Wollega University.

## Declaration of competing interest

The authors declare that they have no competing interests.

## Data Availability

The datasets used and/or analyzed during the current study are available from the Ethiopian Public Health Institute (EPHI) or the corresponding author on reasonable request.
